# Emotion network density in burnout

**DOI:** 10.1186/s40359-021-00670-y

**Published:** 2021-10-30

**Authors:** Tobias R. Spiller, Sonja Weilenmann, Krithika Prakash, Ulrich Schnyder, Roland von Känel, Monique C. Pfaltz

**Affiliations:** 1 Department of Consultation-Liaison Psychiatry and Psychosomatic Medicine, University Hospital Zurich, University of Zurich, Culmannstrasse 8, 8091 Zurich, Switzerland; 2 Department of Psychology, Eastern Michigan University, Ypsilanti, MI USA; 3 University of Zurich, Zurich, Switzerland; 4 Department of Psychology and Social Work, Mid Sweden University, Östersund, Sweden

**Keywords:** Network analysis, Stress, Burnout, Health care worker, Medical students

## Abstract

**Background:**

Health care workers are often affected by burnout, resulting in reduced personal well-being and professional functioning. Although emotional exhaustion is considered a core component of burnout, little is known about the dynamics of emotions and their relation to burnout. We used network analysis to investigate the correlation between the density of a negative emotion network, a marker for emotional rigidity in person-specific networks, and burnout severity.

**Methods:**

Using an ecological momentary assessment design, the intensity of negative emotions of forty-three health care workers and medical students was assessed five times per day (between 6 am and 8 pm) for 17 days. Burnout symptoms were assessed at the end of the study period with the Maslach Burnout Inventory. Multilevel vector autoregressive models were computed to calculate network density of subject-specific temporal networks. The one-sided correlation between network density and burnout severity was assessed. The study protocol and analytic plan were registered prior to the data collection.

**Results:**

We found a medium-sized correlation between the negative emotion network density and burnout severity at the end of the study period *r*(45) = .32, 95% CI = .09–1.0, *p* = .014).

**Conclusions:**

The strength of the temporal interplay of negative emotions is associated with burnout, highlighting the importance of emotions and emotional exhaustion in reaction to occupational-related distress in health care workers. Moreover, our findings align with previous investigations of emotion network density and impaired psychological functioning, demonstrating the utility of conceptualizing the dynamics of emotions as a network.

**Supplementary Information:**

The online version contains supplementary material available at 10.1186/s40359-021-00670-y.

## Background

The strain the SARS-CoV-2 pandemic put on health care workers globally has recently been in the center of the attention of the broader public [1]. However, persistent occupation-related distress, commonly operationalized as burnout, has been common among health care workers long before the pandemic’s beginning [2–4]. Although burnout itself is not considered a mental disorder, it is well known to be related to impaired mental health [5] and altered physiological functioning (e.g., increased sympathetic activity) [6]. Importantly, burnout negatively affects the professional functioning of health care workers as well as the care they provide for their patients [7–9]. Therefore, the prevalence of burnout has become an often monitored metric in health care systems.

Burnout is usually characterized by three dimensions, namely, the experience of energy depletion or emotional exhaustion, a feeling of reduced personal efficacy with regard to one’s work, and a cynical attitude toward the value of one’s occupation (also termed depersonalization [10]. Given that emotional exhaustion constitutes a core dimension of burnout, it does not come as a surprise that emotions and emotional distress play a key role in many conceptualizations of burnout and its pathogenesis [11, 12]. In healthy individuals, emotions are known to vary across time and context, reflecting the capacity to adaptively respond to an ever-changing environment [13, 14]. In individuals affected by burnout, however, negative emotions tend to change slower and persist longer, indicating a diminished capacity to react to environmental challenges [15, 16]. This persistence of emotions is also termed *inertia* and has been commonly measured using the *autocorrelation* of an emotion. Formally speaking, autocorrelation corresponds to the correlation of the intensity of an emotion across two consecutive measurements and is usually studied using repeated assessments at fixed intervals over a longer period of time. Thus, a higher autocorrelation indicates a higher inertia (or persistence), and thus, a slower change of emotions [13]. Besides its association with burnout, inertia of emotions is also associated with impaired psychological functioning in general, but also with specific psychopathologies such as depression [13, 14].

One important limitation of this prior work, however, is that autocorrelation can only study individual emotions or a composite thereof. This is problematic given that emotions do not exist independently but interact with each other [17]. The recently introduced network approach enables the modelling of an individual’s emotional life as a dynamic interplay of emotions, pictured as a network in which nodes correspond to emotions and edges to relationships between them [18]. Hence, the density of an emotion network can take the autocorrelation of emotions as well as their temporal dependency (cross-lagged effect) into account [18, 19]. Consequently, a higher network density is indicative of a more rigid system of emotions, which is less likely to adapt to new environmental challenges. Importantly, the interactions between emotions, i.e., the structure of the network, can vary across individuals. Therefore, network density must be calculated on the basis of person-specific networks and longitudinal data.

To our knowledge, the association between emotion network density and psychological functioning was investigated in three empirical studies so far [19–21]. Pe and colleagues assessed the density of a person-specific 11-node network consisting of positive and negative emotions for each of the 53 individuals with and 51 individuals without depression. They found a higher density in the depressed individuals than in the control group [19]. Notably, this finding held when only the negative emotions were considered for the calculation of network density, but not for network density of positive emotions alone. This finding concurs with the broader literature documenting a stronger relationship between inertia and psychological functioning for negative, but not positive emotions [14]. In a second study, Lydon-Staley and co-authors analysed the data of a daily diary study of 151 adolescents [21]. They reported that greater density of a 4-node network consisting of affective states (happiness, depression, anxiety, and anger), each composed of multiple emotions, was correlated with more symptoms of depression. In a third study, stronger emotion network density of individuals in treatment for depression predicted treatment non-response [20]. However, the assumed stationarity (i.e., that the relationships between the investigated variables do not change during the study period), which is a key assumption of time-series analysis, was violated due to the impact of treatment. A recently published preprint also reported that a higher network density of both negative and positive emotions was predictive for the diagnostic status of anxious and depressed patients when emotions were sampled multiple times a day, but not when assessed only once a day [22].

To our knowledge, no study has investigated the relationship between emotion network density and burnout yet. Such an analysis bears the potential to advance the understanding of interactions among emotions that are assumed to be a perpetuating factor of burnout [11]. Hence, a better understanding of these interactions might also help to clarify why interventions to reduce burnout are helpful in some, but not all individuals [23]. Therefore, this study aimed to test the hypothesis that the density of subject-specific temporal negative emotion networks correlates directly (positively) with burnout severity. Due to the high prevalence and important role of burnout in health care settings, this study focused on health care workers. The study protocol, including the hypothesis, the study design, and the analytic plan, were registered prior to the data collection (and can be found here: https://osf.io/u8fqn).

## Methods

### Participants and procedure

This observational Ecological Momentary Assessment (EMA) study was conducted in Switzerland between October 01, 2019 and July 31, 2020. Due to the restrictions on research with human subjects at the beginning of the SARS-CoV-2 pandemic, the recruitment of participants had to be interrupted in Spring 2020. To be eligible for participation in this study, participants had (a) to be 18 years or older, (b) to live in Switzerland, (c) to work as or to study to become a health care worker, (d) to be in daily contact with patients, and (e) to have access to a smartphone running the iOS or Android operating system. Members or students of the following professions were defined as health care workers: physicians, psychotherapists, nursing staff, physio—, occupational—or speech therapists, paramedics, or medical admin staff with patient contact. Participants were recruited through a study website, posters and flyers distributed at health care facilities and universities, and through personal contacts of the study team members. Subjects willing to participate in this study underwent a telephone interview, during which the details of the study were explained, and eligibility criteria were assessed. Informed consent for participation was obtained from all participants. Participation in this study was reimbursed 20 CHF (approx. USD 20) when at least 50% of the EMA and all questions from the first and the last test day were completed. An additional CHF 20 were reimbursed when at least 80% of the EMA ratings were completed. The study protocol was approved by the ethics committee of the canton Zurich (BASEC-Nr. 2019-01020).

The study lasted a total of 18 days and was split in three phases. On Day 1, cross-sectional data and demographics were assessed. Subsequently, the 17-day long EMA period lasted from Day 2 to the evening of Day 18. Finally, the level of burnout was again assessed cross-sectionally on the evening of Day 18. During the EMA period, participants were prompted to rate their momentary emotions on their smartphone five times a day (between 6am and 6 pm on working days and 8am and 8 pm on weekends). These prompts were delivered pseudorandomly, meaning that each prompt was delivered randomly during a predefined 30-min period. Once prompted, participants had 90 min to provide their ratings. All data was collected using the *LifeData* smartphone application and platform.

The sample size rationale was built upon our hypothesis and methodological considerations. First, the number of observations needed to estimate the planned subject-specific network models cannot readily be obtained. Still, a simulation study indicated that 50 repeated measurements per individual is supposed to be sufficient for such a network consisting of 8 nodes [24]. Thus, based on the analytic procedure (resulting in a loss of 20% of the daily measures due to the exclusion of the overnight lag, see below) and an assumed missing data rate of 20%, we set the length of the EMA period to 17 days, resulting in a total of 85 assessments of emotions (and 54 assessments when assuming a 20% missing rate). Second, we calculated the required sample size to test the correlation between network density and burnout based on the following assumptions: (a) a medium effect size (Spearman’s ρ = 0.3), a conservative assumption given the previously reported large effects [18, 19], (b) a one-tailed, positive correlation, (c) an alpha level of 0.05, and (d) 80% power. This resulted in a target sample size of N = 64, using G*Power [25].

### Measures

Basic demographic data was obtained from all participants including gender (female, male, and other), age (in years), profession (physician, psychotherapist, nursing staff, physiotherapists, occupational therapists, speech therapists, paramedics, and medical admin staff), average weekly work hours, and hours working overtime.

#### Maslach burnout inventory: general survey

We used the Maslach Burnout Inventory-General Survey (MBI-GS) to determine burnout severity. The MBI-GS consists of 16 items, structured in three dimensions. Five items each refer to emotional exhaustion (dimension 1) and depersonalization (dimension 2), whereas professional efficacy (dimension 3) was assessed with six items. All items were rated on an 8-point Likert scale (ranging from 0 indicating never and 7 indicating very strong) with the items assessing professional efficacy being coded reversely. Following common practice (e.g., [26], burnout severity was assessed with a total score, accounting for the different weights of the burnout dimensions and the number of items resulting in a total score ranging from 0 to 6.^1^ The questionnaire was adapted to cover the study period (i.e., 17 days) instead of a full year. Although several German translations of the MBI-GS do exist, none has officially been validated. Nevertheless, the chosen translation has been widely used in previous research (e.g., [27, 28] and demonstrated good internal consistency in this study with Cronbach’s alpha of 0.91 for the MBI-GS total score.

#### Ecological momentary assessment (EMA)

The emotions assessed included four with positive (happy, satisfied, relaxed, being full of energy) and four with negative valence (frustrated, stressed, worried, exhausted). All emotions were assessed using single items (e.g., How frustrated are you at the moment) and a visual analogue scale ranging from 0 (not at all) to 100 (absolutely). Only the emotions with negative valence were included into the primary analysis. However, network density of a network consisting of only positive emotions was calculated in a secondary, not preregistered analysis as well.

### Data analysis

The analytic plan to test our hypothesis was structured in three parts: (a) data preparation, (b) network estimation and density calculation, and (c) correlating density and burnout severity. The three steps followed the analyses as suggested by the developers of these methods [18, 29], and by the authors of previous studies [19, 21, 30].

#### Data preparation and missing values

In a first step, the data obtained with the *LifeData* app was relabelled and reorganized into two datasets, one containing the data of the assessments of the first and last day and the other the EMA data. Next, participants with missing data in the burnout assessment at Day 18 and those with more than 20% missing EMA data were excluded from the analysis. Finally, after the conductance of the analysis outlined below, the density and burnout severity measures were checked for outliers (defined as diverging more than 3 SD from the sample mean), which were excluded from further analysis. All data analysis was conducted in the *R* environment using, among others, the packages *mlVAR* [31], and *tidyverse* [32]. The data and the analytic code are available in the Additional files 1, 2 and 3.

#### Network estimation and density calculation

Person-specific temporal networks were estimated for all individuals using the two-step approach of the multilevel vector auto-regression model (mlVAR; [18, 29]. The mlVAR is an extension of the vector auto-regression model (VAR) to model the individual networks of a group of subjects. A lag-1 VAR model estimates to what extent the rating of each emotion at one timepoint (t_0_) is predicted by the ratings of all emotions (including itself) at the previous rating (t_−1_). Formally, each emotion is regressed on the lagged values of all other emotions (cross-lagged effects) and its own lagged values (autoregressive effects) using a series of univariate regression models [18, 29].

The mlVAR model extends this VAR model into a multilevel modelling framework, in which the average auto- and cross-lagged effects are obtained for the whole sample (fixed effects) but are also allowed to vary across the subjects of a population (random effects; [18, 29]. For this purpose, the variables included into the network were within-subject (i.e., person-mean) centred prior to the analysis. Furthermore, given that the lag, the time between two consecutive prompts, is assumed to be constant, the lag from the first measurement on one day onto the last measurement of the day before was removed from the analysis [18]. Following the definition of previous studies, network density was determined as the average of the absolute values of the cross- and auto-lagged effects of emotions [18, 30, 33]. Therefore, the effects of each subject’s network were extracted, their absolute value was summed and then divided by 20, the total number of the effects in each network. Missing data was handled with listwise deletion.

#### Correlation between density and burnout severity

The correlation between the density of the person-specific networks and burnout severity was quantified using both Pearson's *r* and Spearman's ρ. Both correlations were tested for significance with the help of a one-sided test (assuming a positive correlation). The alpha level was set to 0.05.

## Results

### Demographics

We recruited a total of 69 participants. Of these, 8 had missing data on burnout severity on the last day and 14 had more than 20% missing EMA data and were therefore excluded from further analysis resulting in a final sample size of 47. The sample included mainly women (n = 45, 95.7%) and median age was 25.0 years [*Q*1, *Q*3: 23.0, 30.0]. The sample consisted of 30 (63.8%) medical students, seven (14.9%) psychotherapists, four (8.5%) physicians, two (4.3%) nurses, four (8.5%) participants with other professions (e.g., physio—, occupational—or speech therapists). The Median number of EMA observations provided by the participants was 77 [90.6%; *Q*1, *Q*3: 72.5, 79] out of the 85 prompts received, indicating high compliance with the sampling protocol.

### Network density and burnout severity

**Fig. 1:**
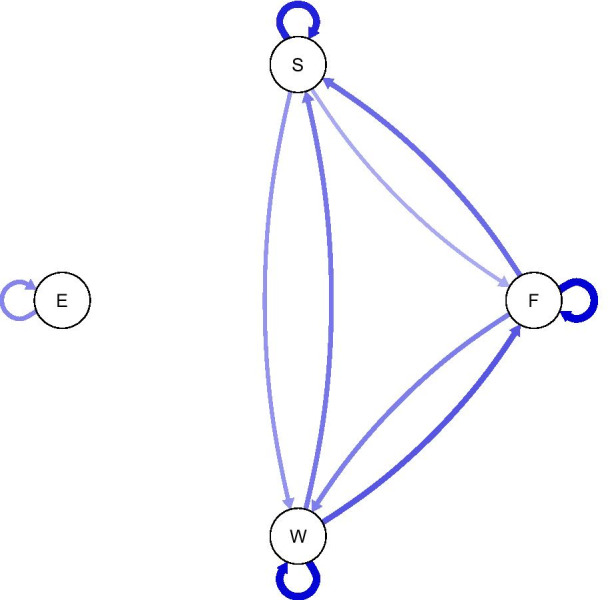
Visualization of the group-level emotion network. Edges indicate relationships between two emotions with one emotion at t_0_ being predicted by the value of the other at t_−1_. The thickness of the edges corresponds to their strength, blue edges represent positive, red edges negative relationships. S = stressed, E = exhausted, F = frustrated, W = worried

**Fig. 2:**
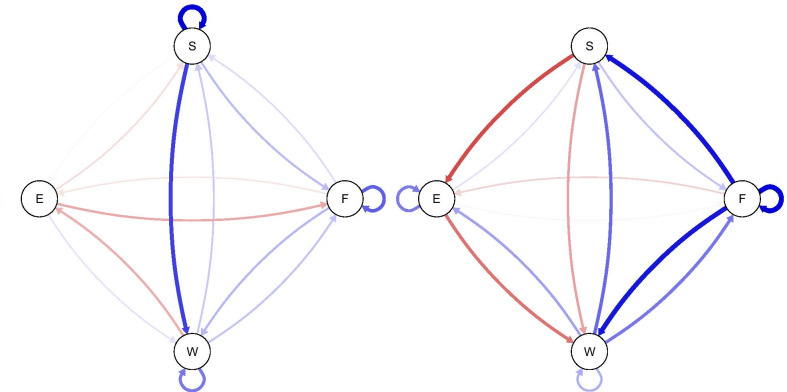
Visualization of two person-specific emotion network of participants. Visualization of two person-specific emotion network of participants number 8 and 22. Edges indicate relationships between two emotions with one emotion at t_0_ being predicted by the value of the other at t_−1_. The thickness of the edges corresponds to their strength, blue edges represent positive, red edges negative relationships. S = stressed, E = exhausted, F = frustrated, W = worried

Figure 1 depicts the temporal network of the full sample, showing only significant edges. All edges were positive. Moreover, this was also the only relationship of exhaustion with the remaining three nodes of the network. Figure 2 shows two examples of person-specific networks. Here, the number of relationships between the nodes as well as their direction and strength varied greatly across the investigated individuals.

**Fig. 3:**
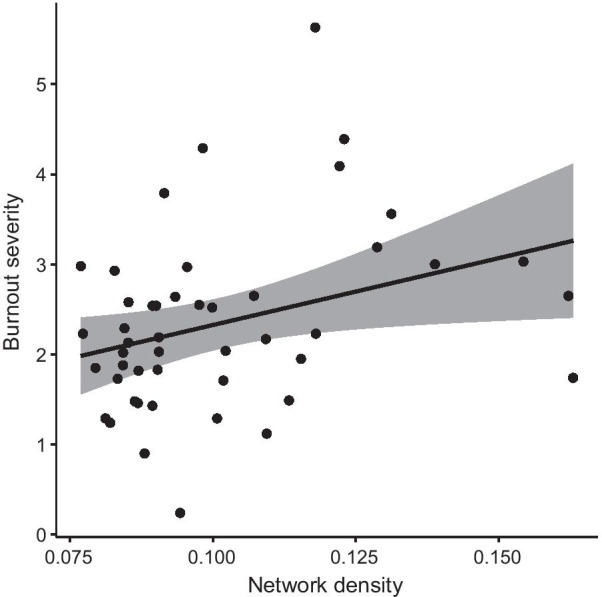
Correlation between network density and burnout severity. Scatterplot with the x-axis denoting the network density (ranging from 0 to 1) and the y-axis denoting burnout severity (ranging from 0 to 6). The black line indicates the correlation, the grey area the confidence intervals

Median network density was calculated as 0.093 [Q1, Q3: 0.086, 0.111]. The mean burnout severity was 2.19 [Q1, Q3: 1.74, 2.79]. Results of the one-sided Pearson correlation analysis indicated a direct association between network density and severity of burnout (*r*(45) = 0.32, 95% CI = 0.09–1.0, *p* = 0.014). The same was observed in the one-sided Spearman correlation analysis (*s*(45) = 0.36, *p* = 0.006). A scatterplot indicating the individual participants’ network density and burnout severity is shown in Fig. 3.

Our secondary, not preregistered analysis estimating positive emotion networks revealed an average network density of 0.096 [Q1, Q3: 0.089, 0.113]. Both correlation analyses showed non-significant results: Pearson correlation: (*r*(45) = 0.14, 95% CI =  − 0.09 to 1.0, *p* = 0.143) and Spearman correlation (*s*(45) = 0.19, *p* = 0.106).

## Discussion

In this study, we examined the extent to which the temporal dependencies of negative emotions relate to burnout in health care workers and medical students using a network approach. In line with our preregistered hypothesis, we found that the density of person-specific temporal networks of negative emotions was directly correlated with burnout. Our findings are in accordance with previous research on network density and psychological functioning [19–22] as well as with two studies on emotional inertia in burnout [15, 16].

Regarding the role of emotions in burnout, our results are corroborated by a recently published study demonstrating a relationship between exhaustion and the inertia of negative emotions [16]. Interestingly, the authors reported that exhaustion predicted inertia a month later, whereas the opposite was not the case, indicating that the feeling of exhaustion precedes an increased inertia of negative emotions. This is partly in contrast to our study, in which network density correlated with burnout. Nonetheless, our findings highlight that burnout is associated with changes in the interplay of negative emotions and their increased persistence. Moreover, an increased persistence of a feeling of emotional exhaustion, frustration, or tiredness does operationalize one of the three dimensions of burnout itself [34]. Thus, increased network density might not only be associated with burnout but might constitute a model to describe the pathogenesis of burnout itself, or at least one of its dimensions. Nevertheless, future research is warranted to reveal the temporal sequence of the changes in burnout severity and negative emotion network density. Additional evidence in line with our findings stems from a study that used heart rate variability (HRV) as a psychophysiological measure for emotion regulation capacity, with lower HRV indicating lower emotion regulation capacity [35]. The authors reported that workers with lower HRV showed higher persistence of negative emotions at work [15]. Notably, burnout has repeatedly been shown to be related to lower HRV in working populations [36, 37]. Still, the relationships between HRV, burnout and network density remain to be investigated.

Our finding of a relationship between network density of person-specific temporal emotion networks and burnout severity is in agreement with similar observations in studies in depressed adults [19, 20], adolescents [21], and adults with depression and anxiety [22]. Our study therefore successfully expanded the range of phenomena associated with increased network density. It might be that network density, similarly to emotional inertia, is transdiagnostically related to reduced psychological functioning [13, 14]. However, in contrast to previous studies, our primary analysis was restricted to negative emotions. While one study found that the density of positive emotions was not related to depression [19], the three others did either only investigate the density of a mixed valence emotion network [20, 21] or found evidence for a predictive value of both, negative and positive emotion network density [22]. To address this limitation, we conducted a secondary, not preregistered analysis of the relationship between burnout and the density of a positive emotion network density, again assuming a positive correlation and following otherwise the same analytic protocol. In our study, the correlation was not significant.

### Limitations

This study’s findings are subject to several limitations. First, due to the restrictions on research with human subjects at the beginning of the SARS-CoV-2 pandemic, we were not able to recruit enough participants to meet the target sample size. Second, our sample mainly consisted of (white and young) women (n = 45, 95.7%) limiting the generalizability of our findings to more diverse populations. Furthermore, more than half of the participants were medical students (n = 30, 63.8%), limiting the sample’s representativeness of health care professionals in general. The underrepresentation of health care professionals in our sample might be the result of their limited resources to participate in studies due to their busy schedules. In addition, the additional strain of the SARS-CoV-2 pandemic might have also limited the willingness of health care professionals to participate in laborious studies. Third, we relied on the same measurement practices like previous work (e.g., [18, 19] using single items to assess the intensity of emotions. Still, these measures were not validated. However, the design of measures specifically for an EMA context and their validation is still in its infancy [38], the advancements are rapid and future studies should aim to incorporate these developments (e.g. [39]. Fourth, the scope of our network was limited to only four negative emotions. The inclusion of more emotions in future studies could cover a larger spectrum of negative emotions but will in turn require more observations of these emotions to reach similar power. Fifth, the timescale of the MBI-GS was adopted to cover the period during which emotions were measured. Again, this adaptation as well as the translation were not validated in advance. At least, the internal consistency of the burnout scale in this study was found to be good. Last but not least, a recent simulation study questioned whether VAR models based on EMA data can be used to recover the mircodynamics of emotions [40]. Still, whether this fundamentally limits the use of VAR models remains to be discussed [41]. The limitations of this study are counterbalanced by the preregistration and the open data, which constitute two major strengths.

The investigation of the change of emotion dynamics, is an important avenue for future research. On the basis of the theoretical considerations, one could assume that there are two “stable” states, a healthy one, and one characterized by feelings of burnout. In both states, the dynamics of negative emotions in individuals are fixed but as implied by our findings, are stronger (i.e., the network is denser) in a state of burnout. Consequently, the strength of the dynamics itself changes when an individual begins to develop burnout. While this cannot be investigated with a mlVAR model, as it assumes the stationarity of these dynamics, new methods to capture this change have recently been introduced [18, 24, 41]. A different, but equally important topic for future research concerns the length of the interval between two consecutive prompts. Notably, one study found emotion network density to be predictive of depression and anxiety when participants’ emotions were sampled at 90-min intervals, but not when sampled only once daily, highlighting the importance of the length of the interval between the two assessments [22]. In the context of burnout, however, intensive sampling rates pose several challenges. First, the strain of high sampling rates could directly harm participants, increasing their risk for burnout. Importantly, overcommitment, which likely increases the willingness to participate in a study, is an important risk factor for poor mental health and burnout [42]. Thus, the well-being of participants should be monitored closely. Second, highly intensive sampling rates will likely prevent the busiest workers from participating and therefore exclude the population most at risk for burnout. One possibility to overcome this limitation is the use of psychophysiological measures like HRV as a proxy for the adaptive capacity of emotions. Still, the associations between HRV network density and burnout remain to be investigated first. This study’s findings do not directly translate into clinical interventions for the prevention or treatment of burnout. Nevertheless, knowledge about the network structure of negative emotions is prerequired to design emotion-focused, network-based interventions for burnout [43]. Such interventions could target specific negative emotions to reduce their severity which would then, assuming causal interactions between the nodes, be expected to result in a reduction in the overall severity across multiple negative emotions [44]. Moreover, it might be worthwhile to assess whether third wave cognitive behavioral approaches that focus on client’s attitudes towards their emotional states (Hayes & Hofmann, 2017) impact the density of the network of negative emotions [45]. Finally, the density of a network of negative emotions assessed with EMA might be used to track the effectiveness of preventive or therapeutic interventions for burnout.

## Conclusion

In this preregistered EMA study, we found a positive correlation between the density of person-specific temporal negative emotion networks and burnout severity in health care workers and medical students. The medium size of this correlation emphasizes the importance of the interplay of negative emotions in burnout. In addition, our findings highlight the utility of a network approach-based hypothesis for the understanding of the dynamics of emotions in burnout.

## Supplementary Information

**Additional file 1**. Data file containing demographic data and data collected at the last day of the study; data separated by “;”

**Additional file 2**. Data file containing EMA data; data separated by “;”

**Additional file 3**. *R* code used in the presented analyses

## Data Availability

The datasets analyzed during the current study and the analytic code are available in the Additional files 1, 2 and 3.

## Abbreviations

CHF: Swiss francs
EMA: Ecological momentary assessment
HRV: Heart rate variability
MBI-GS: Maslach Burnout Inventory-General Survey
mlVAR: Multilevel vector auto-regression model
SARS-CoV-2: Severe acute respiratory syndrome coronavirus type 2
USD: US Dollar
VAR: Auto-regression model

## Footnotes

1 The total score was defined as (0.40 × (the average of the emotional exhaustion subscale) + 0.30 × (the average of the depersonalization subscale) + 0.30 × (the average of the reversely coded personal accomplishments subscale).
